# YTHDC1 mitigates ischemic stroke by promoting Akt phosphorylation through destabilizing *PTEN* mRNA

**DOI:** 10.1038/s41419-020-03186-2

**Published:** 2020-11-13

**Authors:** Zhaolong Zhang, Qiuhan Wang, Xiaolong Zhao, Liming Shao, Guoping Liu, Xuan Zheng, Lingling Xie, Yan Zhang, Chengjian Sun, Rui Xu

**Affiliations:** Department of Interventional Radiology, the Affiliated Hospital of Qingdao University, Jiangsu Road 16, Qingdao, 266000 Shandong China

**Keywords:** Cell death in the nervous system, Stroke, Stroke

## Abstract

YTH Domain Containing 1 (YTHDC1) is one of the m^6^A readers that is essential for oocyte development and tumor progression. The role of YTHDC1 in neuronal survival and ischemic stroke is unknown. Here, we found that YTHDC1 was unregulated in the early phase of ischemic stroke. Knockdown of YTHDC1 exacerbated ischemic brain injury and overexpression of YTHDC1 protected rats against brain injury. Mechanistically, YTHDC1 promoted *PTEN* mRNA degradation to increase Akt phosphorylation, thus facilitating neuronal survival in particular after ischemia. These data identify YTHDC1 as a novel regulator of neuronal survival and modulating m^6^A reader YTHDC1 may provide a potential therapeutic target for ischemic stroke.

## Introduction

Ischemic stroke is one of the leading causes of death worldwide. Many biochemical and molecular events including glutamate excitotoxicity, oxidative stress, apoptosis, and inflammation have been identified to be involved in the ischemic neuronal injury^1,2^. These events are intricately regulated in the transcriptional, post-transcriptional, and post-translational levels^3–5^. In the past few decades, hundreds of agents targeting the above pathophysiological mechanisms of acute ischemic stroke have failed in clinical trials^6,7^. Considering the limited efficacy of thrombolysis and mechanical thrombectomy in the treatment of ischemic stroke^8^, new agents are now required.

Control of mRNA metabolism is critical for managing the quantity of gene expression^9^. The post-transcriptional regulation of mRNA can help cells respond rapidly to external stimuli and stresses^10,11^. N6-methyladenosine (m^6^A) is the most abundant and reversible modification of RNA and is dynamically regulated by m^6^A “writers”, “readers”, and “erasers”. The cellular functions of m^6^A have been connected directly to the splicing, export, stability, and translation of mRNA, and consequently have been associated with enormous functions, such as cell proliferation, migration, and differentiation^12–14^. Although a previous study demonstrated that transient focal cerebral ischemia could alter the m^6^A modification of mRNAs and lncRNAs^15^, the role of m^6^A modification in cell survival especially in ischemia-induced neuronal survival is still unclear.

YTHDC1 is one of the five known mammalian YTH domain-containing m^6^A “readers” (YTHDF1, YTHDF2, YTHDF3, YTHDC1, and YTHDC2), a group of proteins which can recognize m^6^A modification^16^. As the cytoplasm-localized YTH domain family proteins, YTHDF1, YTHDF2, and YTHDF3 can modulate cell survival, proliferation, and migration via facilitating RNA degradation^17–20^. YTHDC1 is observed to be mainly localized in the nucleus, can shuttle into the cytoplasm, and is essential for pre-mRNA splicing^21^, RNA export ^22,23^, and mRNA destabilization^24^. So far, the role of YTHDC1 in cell survival and ischemic stroke is not understood.

In this study, we found that YTHDC1 was unregulated in the early phase of ischemic stroke. Knockdown of YTHDC1 exacerbated ischemic brain injury and overexpression of YTHDC1 alleviated brain ischemia. At the cellular level, YTHDC1 promoted neuronal survival through maintaining Akt phosphorylation. Notably, YTHDC1 promoted *PTEN* mRNA degradation to increase Akt phosphorylation. Our data elucidate a new role of YTHDC1 in brain injury and indicate the potential involvement of m^6^A modification in the regulation of ischemic stroke.

## Materials and methods

### Rats and focal cerebral ischemia

Adult male Sprague-Dawley rats (200–250 g) were housed with three rats per cage on a 12 h light/dark cycle in a temperature-controlled room with free access to water and food. All animal experiments were conducted in compliance with National Institutes of Health guidelines and were approved by the institutional animal care and use committee of Qingdao university.

The suture occlusion technique was used to induce transient focal cerebral ischemia according to the previous reports^25,26^. Male SD rats weighing 200–250 g were anesthetized using 4% isoflurane in 70% N_2_O and 30% O_2_ with a mask. A midline incision was made in the neck, the left external carotid artery (ECA) was carefully exposed and dissected, a monofilament nylon suture with a diameter of about 0.22 mm was inserted from the ECA into the internal carotid artery, and the left middle cerebral artery (MCA) was blocked. After occlusion for 90 minutes, the suture was removed for reperfusion, and ECA was ligated to close the wound. Sham-operated rats underwent the same surgery except for suture insertion. Rats were maintained on top of a warming pad (RWD, 69003) during the above procedures. The breathing machine was used to monitor the respiration of rats. The rats were returned to a heated cage during the recovery phase with free access to food and water.

In total, MCAO was successfully induced in 99 rats that were used in further experiments and 9 mice died because of unsuccessful MCAO within 24 h after reperfusion which were excluded from the experiments.

### 2,3,5-triphenyltetrazolium chloride (TTC) staining and infarct volume measurement

We harvested the brain at 24 h after transient middle cerebral artery occlusion (tMCAO) and performed TTC staining. The brain was placed in a cooled matrix and cut into 2 mm coronal slices. The brain sections were placed in a 10 cm petri dish and incubated for 30 min at 37 °C with 2% TTC in phosphate buffered saline. Sections were then fixed in 4% paraformaldehyde at 4 °C for 24 h. Viable brain tissue was stained red, whereas a pale gray color indicated infarcted tissue. All image acquisition, processing and analysis were performed blindly. Image analysis software (Image-Pro Plus Version 6.0, USA) was used to analyze the scanned images. Percent of infarct volume was calculated as previously described^27,28^.

### Neurological severity score analysis

At 24 h after induction of MCAO, neurological deficits of rats were tested using the following 6-point scale according to previous reports^29^: no neurological deficits (0 points); flexion of the contralateral torso and forearmon lifting the animal by the tail (1 point); circling to the contralateral side but normal posture at rest (2 points); leaning to the contralateral side (3 points); and no spontaneous motor activity (4 points); stroke-related death (5 points). Randomization was used to assign samples to the experimental groups, and to collect and process data. The experiments were performed by investigators blinded to the groups for which each animal was assigned.

### Primary cortical neuron culture and OGD treatment

The cortical neurons were prepared from the cortex of embryonic day 17 (E17) of rats embryos as described^27^. The dissociated cortical neurons were suspended in plating medium (Neurobasal medium, 2% B-27 supplement, 0.5% FBS, 0.5 mM L-glutamine, and 25 mM glutamic acid) and plated on poly-D-lysine-coated dishes. Half of the plating medium was removed and replaced with maintenance medium (Neurobasal medium, 2% B-27 supplement, and 0.5 μM L-glutamine) after 1 day in culture. From then on, the culture medium was changed every 3 days. After 12 days, the cultured neurons were used for further experiments.

Oxygen-glucose deprivation (OGD) challenge was performed as previously described^27,30^. Neurons were transferred to deoxygenated glucose-free extracellular solutions (116 mM NaCl, 5.4 mM KCl, 0.8 mM MgSO_4_, 1.0 mM NaH_2_PO_4_, 1.8 mM CaCl_2_, and 26 mM NaHCO_3_); introduced into a dedicated chamber (Plas-Labs, Lansing, USA) and held in 85% N_2_/10% H_2_/5% CO_2_ at 37 °C for 60 min. The medium was then replaced with fresh maintenance medium containing the appropriate concentration of reagents for the indicated times during recovery in a 95% O_2_/5% CO_2_ incubator. Control cultures were first transferred to another extracellular solution (116 mM NaCl, 5.4 mM KCl, 0.8 mM MgSO_4_, 1.0 mM NaH_2_PO_4_, 1.8 mM CaCl_2_, 26 mM NaHCO_3_, and 33 mM glucose), and humidified at 37 °C for 60 min in 95% O_2_/5% CO_2_. The entire medium was then replaced with fresh maintenance medium in a 95% O_2_/5% CO_2_ incubator at 37 °C.

### Lentivirus in vitro infection and in vivo administration methods

#### Lentivirus preparation

For YTHDC1 overexpression experiments, cDNAs of rat YTHDC1 gene were cloned into the pCDH-CMV-MCS-EF1-GFP lentiviral vector. To knockdown YTHDC1, the shRNA sequence: CGACCAGAAGATTATGATA was selected to construct the lentiviral vector GV493 (hU6-MCS-CBh-gcGFP-IRES-puromycin, GeneChem, Shanghai, China). The virus was packaged in the HEK 293T cell line according to the manufacturer’s instructions, and after transfection for 48 h, viral culture supernatants were harvested and concentrated.

#### In vitro infection

Primary cortical neurons were infected with the concentrated lentivirus. Two days after the infection, the infected neurons were used for further analysis.

#### In vivo administration

Lentiviruses were stereotaxically injected into the cortex at four sites on the left as follows as described previously^29^: site 1, 0.3 mm anterior to bregma, lateral 3 mm to midline; depth, 2 mm to skull surface; site 2, 0.3 mm anterior to bregma, lateral 3 mm to midline; depth, 5 mm to skull surface; site 3, 0.3 mm anterior to bregma, lateral 5 mm to midline; depth, 3 mm to skull surface; site 4, 0.3 mm anterior to bregma, lateral 5 mm to midline; depth, 6 mm to skull surface. The injection was performed randomly. After injection for 14 days, MCAO was performed in the left side of brain.

### Neuron viability assays

CCK8 assay was performed as described below. Cell survival was assayed by Cell Counting Kit-8 (Solarbio) based on the manufacturer’s instructions. Cortical neurons were plated at a density of 1 × 10^5^ cells per well in 24-well plates. After virus transfection or treatment, CCK-8 solution was added into each well, followed by incubation for 1–4 h. Cell viability was determined by measuring the OD at 450 nm. Percent over control was calculated as a measure of cell viability.

LDH release was measured using the CytoTox 96 Cytotoxicity Kit according to the manufacturer’s instructions (Promega, USA). The levels of maximal LDH release levels were measured by treating cells with 10× lysis solution to yield complete lysis of the cell. Absorbance data were obtained using a 96-well plate reader (Molecular Devices, USA) at 490 nm. LDH release (%) was calculated by calculating the ratio of experimental LDH release to maximum LDH release according to the manufacturer’s instructions.

### Quantitative real-time PCR analysis

Total RNA was extracted from cultured cells with RNAfast200 purification kit (Fastagen), and reverse-transcribed with the ReverTra Ace® qPCR RT Master Mix with gDNA Remover (TOYOBO). Real-time PCR was performed on the Biorad 96 touch machine (Bio-Rad) with 2×RealStar Green Power Mixture (GenStar). The sequences of quantitative PCR primers for the genes examined are listed in Supplementary Table 1.

For RNA stability analysis, ActD was added at 1 μg/ml, then 0, 2, 4, 6, and 8 h after the ActD treatment, the cells were harvested to isolate total RNA, and RNA quantification was done by qRT-PCR.

### RNA immunoprecipitation (RIP)

Cells were harvested and lysed with lysis buffer (50 mM Tris-HCl, pH 7.0, 150 mM NaCl, 1 mM MgCl_2_, 0.05% NP-40, 1 mM phenylmethylsulfonyl fluoride, 10 mM Ribonucleoside Vanadyl Complex) supplemented with 140 Uml^−1^ of SUPERase•In (Thermo Fisher Scientific) and protease inhibitor cocktail. The clarified lysates were incubated with anti-YTHDC1 antibody or IgG overnight at 4 °C. Then 30 µl of protein A/G magnetic beads (Thermo Fisher Scientific) were added and incubated for 2 h at 4 °C. After four washes using lysis buffer, the RNA on beads was extracted using TRIZOL reagent and qRT-PCR was performed. For comparison of the binding ability between RNA and proteins, relative enrichment was first normalized to input and then analyzed by comparison with the data from the sample immunoprecipitated with anti-YTHDC1 and anti-IgG.

### Statistical analysis

The statistical analysis was performed using GraphPad Prism software. All experiments were performed for three or more times unless otherwise indicated. All data were shown as means ± S.E.M. To compare the statistical significance of two groups, Student’s *t*-test was used; differences between groups were determined using ANOVA.

## Results

### YTHDC1 expression is upregulated after ischemia

**Fig. 1:**
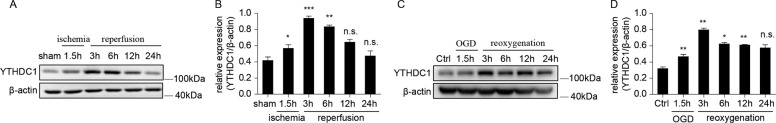
YTHDC1 is induced after ischemia. **A**, **B** Representative immunoblot (**A**) and quantification (**B**) of YTHDC1 in tissue extracts of rat brains upon 90-min MCAO followed by various time points of reperfusion. β-actin served as a loading control. **C**, **D** Representative immunoblot (**C**) and quantification (**D**) of YTHDC1 in cultured primary cortical neurons subjected to OGD/R for the indicated time points. The data are means ± S.E.M., for all panels: *P < 0.05, **P < 0.01, ***P < 0.001 by one-way ANOVA analysis; n.s., no significance. All data are representative of or combined from three independent experiments.

To investigate the role of YTHDC1 after ischemia stroke, we first examined the expression pattern of the five known m^6^A “readers” in a rat model of cerebral ischemia/reperfusion (I/R). Rats are subjected to tMCAO for 1.5 h, followed by reperfusion. Notably, the expression of YTHDF1, YTHDF2, and YTHDF3 were reduced in the ipsilateral cortex after reoxygenation (Fig. S1A, B), whereas the expression of YTHDC1 (Fig. 1A, B) and YTHDC2 (Fig. S1A, B) was remarkably increased in the ipsilateral cortex after 3 h of reoxygenation, remained elevated till 12 h of reoxygenation and then began to decline 24 h post-reoxygenation, compared to its expression in the sham control group (Fig. 1A, B). Consistent with the in vivo results, YTHDC1 expression was also observed to reach a maximum at 3–6 h after reoxygenation after oxygen-glucose deprivation (OGD) in the primary cortical neuron cell cultures (Fig. 1C, D). These results indicate that YTHDC1 is induced in neurons after ischemic stroke.

### Knockdown of YTHDC1 exacerbates ischemic brain injury

**Fig. 2:**
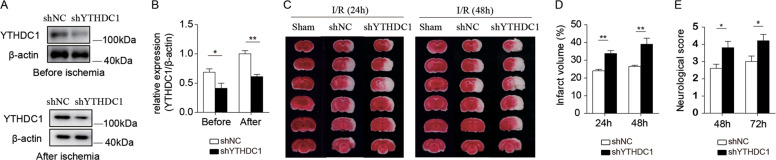
Knockdown of YTHDC1 exacerbates ischemic brain injury. **A** Representative immunoblot and quantification (**B**) of YTHDC1 in corresponding rat brain tissues before ischemia or after ischemia after injection with shNC or shYTHDC1 lentivirus for 2 weeks. β-actin served as a loading control. **C** TTC-stained sections from shNC or shYTHDC1-injected rats at 24 and 48 h after tMCAO. **D**, **E** Quantification of the infarct volume (**D**) and neurological deficit score (**E**) after MCAO. *n* = 5–7. The data are means ± S.E.M., for all panels: *P < 0.05, **P < 0.01, ***P < 0.001 by two-way ANOVA analysis. All data are representative of or combined from at least three independent experiments.

As stroke induced neuronal expression of YTHDC1, we examined whether knockdown of YTHDC1 influenced ischemic brain injury. The left hemisphere of rat was stereotactically injected with lentiviral virus ShYTHDC1 and ShNC, and tMCAO was performed 2 weeks later. Western blot analysis showed that the expression of YTHDC1 was significantly lower in brains injected with ShYTHDC1 virus than brains injected with ShNC virus (Fig. 2A, B). Notably, rats injected with ShYTHDC1virus exhibited increased infarct volume (Fig. 2C, D) and impaired neurological function (Fig. 2E) than rats injected with ShNC virus. These results suggest that knockdown of YTHDC1 exacerbates ischemic brain injury.

### Overexpression of YTHDC1 protects rats against brain ischemic injury

**Fig. 3:**
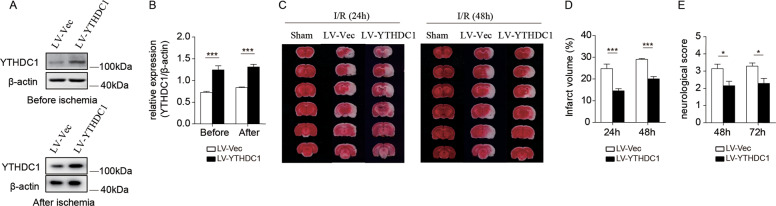
Overexpression of YTHDC1 mitigates ischemic brain injury. **A** Representative immunoblot and quantification (**B**) of YTHDC1 in corresponding rat brain tissues before ischemia or after ischemia after injection with LV-Vec or LV-YTHDC1 lentivirus for 2 weeks. β-actin served as a loading control. **C** TTC-stained sections from LV-Vec or LV-YTHDC1-injected rats at 24 and 48 h after MCAO. **D**, **E** Quantification of the infarct volume (**D**) and neurological deficit score (**E**) after MCAO. *n* = 5–7. The data are means ± S.E.M., for all panels: *P < 0.05, **P < 0.01, ***P < 0.001 by two-way ANOVA analysis. All data are representative of or combined from at least three independent experiments.

Given that knockdown of YTHDC1 worsened I/R-induced cerebral injury, we further investigated whether forced overexpression of YTHDC1 would promote neuronal survival. Local injection of LV-YTHDC1 led to an obvious increase of YTHDC1 abundance compared with LV-Vec-injected controls (Fig. 3A, B). As predicted, overexpression of YTHDC1 alleviated brain injury (Fig. 3C, D) and neurological deficits (Fig. 3D). These data indicate that forced overexpression of YTHDC1 mitigates ischemic stroke.

### YTHDC1 facilitates post-ischemic neuronal survival

**Fig. 4:**
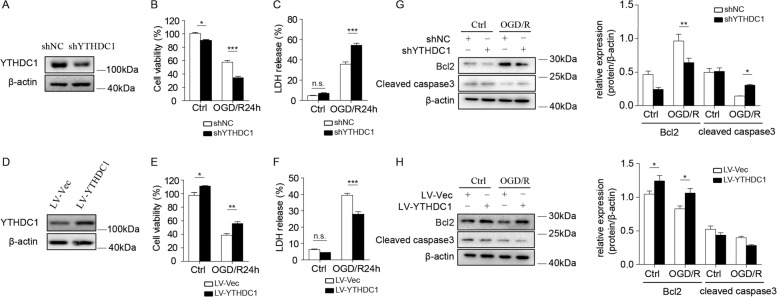
Lentivirus-mediated overexpression or knockdown of YTHDC1 regulates neuronal survival after OGD. **A** Representative immunoblot of YTHDC1 in primary cortical neurons after transfection with shNC or shYTHDC1 lentivirus for 2 days. β-actin served as a loading control. **B**, **C** Cell viability (**B**) or LDH release (**C**) was assessed and quantified for transfected neurons subjected to OGD/R for 24 h or under no treatment conditions (Ctrl). The primary cultured neurons were transfected with shNC or shYTHDC1 lentivirus for 2 days before cell viability or LDH release assay. **D** Representative immunoblot of YTHDC1 in primary cortical neurons after transfection with LV-Vec or LV-YTHDC1 lentivirus for 2 days. β-actin served as a loading control. **E**, **F** Cell viability (**E**) or LDH release (**F**) was assessed and quantified for transfected neurons subjected to OGD/R for 24 h or under no treatment conditions (Ctrl). The primary cultured neurons were transfected with LV-Vec or LV-YTHDC1 lentivirus for 2 days before cell viability or LDH release assay. **G** Representative immunoblot and quantification of Bcl2 and cleaved caspase 3 (cleaved cas3) in lysates of shNC or shYTHDC1-transfected neurons subjected to OGD/R for 3 h or under normal conditions (Ctrl). β-actin served as a loading control. **H** Representative immunoblot and quantification of Bcl2 and cleaved caspase 3 (cleaved cas3) in lysates of LV-Vec or LV-YTHDC1-transfected neurons subjected to OGD/R for 3 h or under normal conditions (Ctrl). β-actin served as a loading control. The data are representative of three independent experiments. The data are means ± S.E.M., for all panels: *P < 0.05, **P < 0.01, ***P < 0.001 by two-way ANOVA analysis. All data are representative of or combined from at least three independent experiments.

Considering the protective role of YTHDC1 against ischemic brain injury, we investigated whether YTHDC1 exerts a direct effect on neuronal survival. We infected primary cortical neurons of rats with either ShNC or ShYTHDC1 virus, which significantly reduced YTHDC1 expression (Fig. 4A). Notably, YTHDC1-slienced neurons were more susceptible to OGD-induced cell death compared with corresponding controls, as evidenced by reduced cell viability and increased LDH release (Fig. 4B, C). Transfection with LV-YTHDC1 enhanced the expression of YTHDC1 in relative to neurons transfected with LV-Vec (Fig. 4D). In consistent with the impacts of YTHDC1 knockdown on neuronal survival, YTHDC1 overexpression (LV-YTHDC1) rendered the neurons more resistant to OGD-induced cell death (Fig. 4E, F).

To further validate the protective role of YTHDC1 in reducing cell apoptosis, we tested the expression of the antiapoptotic protein Bcl2 and pro-apoptotic protein cleaved caspase-3. Knockdown of YTHDC1 reduced the expression of anti-apoptotic protein Bcl2 and increased the expression of cleaved caspase 3 (Fig. 4G), and the results were reversed after overexpression of YTHDC1 (Fig. 4H), which confirmed the protective effect of YTHDC1 on OGD-induced neuronal death. Taken together, YTHDC1 potentiates neuronal survival after OGD, which is consistent with the in vivo results.

### Silencing of YTHDC1 reduces the phosphorylation of Akt via upregulating PTEN expression after OGD

**Fig. 5:**
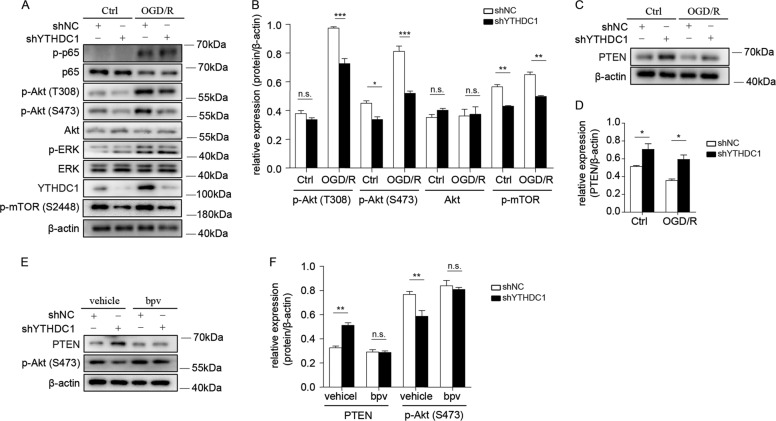
Silencing of YTHDC1 reduces the phosphorylation of Akt via upregulating PTEN expression. **A**, **B** Representative immunoblot (**A**) and quantification (**B**) of the phosphorylated (p-) or total proteins in lysates of shNC or shYTHDC1-transfected neurons subjected to OGD/R for 3 h or under normal conditions (Ctrl). β-actin served as a loading control. **C**, **D** Representative immunoblot (**C**) and quantification (**D**) of PTEN in lysates of shNC or shYTHDC1-transfected neurons subjected to OGD/R for 3 h or under normal conditions (Ctrl). **E** Representative immunoblot and quantification of PTEN and p-Akt in lysates of vehicle or bpv-treated neurons subjected to OGD/R for 3 h. β-actin served as a loading control. The data are representative of three independent experiments. The data are means ± S.E.M., for all panels: *P < 0.05, **P < 0.01, ***P < 0.001, by two-way ANOVA analysis; n.s., no significant. All data are representative of or combined from at least three independent experiments.

To investigate the downstream signaling pathways that participate in YTHDC1-mediated neuronal survival, we tested several potential signaling pathways involved in OGD-induced neuronal survival including NFκB, Akt and ERK signaling pathways^27,31,32^. Early reoxygenation (3 h) after OGD induced an obvious increase of phosphorylation of ERK (p-ERK), p65 (p-p65), and Akt at Ser473 (p-Akt-S473) as well as Thr308 site (p-Akt-T308). Notably, YTHDC1 knockdown robustly decreased the phosphorylation of Akt at both of these two sites after OGD and the abundance of Akt was not altered (Fig. 5A, B). In addition, knockdown of YTHDC1 also moderately downregulated Akt phosphorylation at normal conditions (Fig. 5A, B), which is consistent with the results of Fig. 4. To further test which downstream pathway of Akt signaling was affected after knockdown of YTHDC1, we tested the main pathways downstream of Akt that regulates cell survival including Bcl2 and mTOR activation. Notably, the expression of anti-apoptotic protein Bcl2 (Fig. 4G) and phosphorylation of mTOR (Fig. 5A) was decreased after knockdown of YTHDC1, which confirmed the protective effect of YTHDC1 on OGD-induced neuronal death.

As previous studies have reported that PTEN could inhibit Akt phosphorylation^33–36^, we wondered whether YTHDC1 promoted Akt phosphorylation via downregulating PTEN expression. As speculated, knockdown of YTHDC1 slightly increased PTEN expression in normal conditions, and this effect was more prominent after reoxygenation in neurons (Fig. 5C, D). In addition, reducing PTEN expression in shYTHDC1-transfected neurons by treating cells with PTEN inhibitor bpv^37,38^ restored the phosphorylation of Akt (Fig. 5E, F). Therefore, YTHDC1 may promote neuronal survival especially after OGD through enhancing PTEN expression.

### YTHDC1 decreases *PTEN* mRNA stability in neurons after OGD

**Fig. 6:**
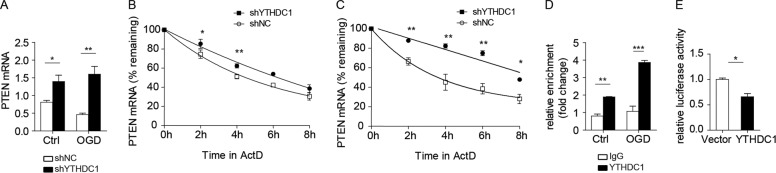
YTHDC1 binds *PTEN* mRNA to decrease its stability. **A** Quantitative PCR analysis of PTEN in shNC or shYTHDC1-transfected neurons subjected to OGD for 1 h or under normal conditions (Ctrl). β-actin served as a loading control. **B** The qRT-PCR analysis of *PTEN* mRNA levels in shNC or shYTHDC1-transfected neurons under normal conditions after ActD (1 µg/ml) treatment for the indicated times. **C** The qRT-PCR analysis of *PTEN* mRNA levels in shNC or shYTHDC1-transfected neurons subjected to ActD (1 µg/ml) treatment for the indicated times after OGD for 1 h. **D** RIP qRT-PCR analysis of PTEN and β-actin in YTHDC1-RNA complexes. Data are presented relative to control immunoprecipitation of protein-RNA with immunoglobulin G (IgG). **E** Dual-luciferase reporter assay in lysates of PC12 cells transfected with luciferase reporter plasmids for 3′-UTR of *PTEN* plus YTHDC1 or Vector. Results are relative to Renilla luciferase activity. The data are means ± S.E.M., for all panels: *P < 0.05, **P < 0.01, ***P < 0.001. **B**, **C**, **E** used student’s *t* test; **A** and **D** used two-way ANOVA analysis. All data are representative of or combined from at least three independent experiments.

To investigate the molecular mechanism of YTHDC1-mediated PTEN downregulation, we first tested the mRNA level of PTEN. qRT-PCR showed that knockdown of YTHDC1 increased the abundance of *PTEN* transcripts in neurons either under normal conditions or early after reoxygenation (3 h) (Fig. 6A). The increase in the amount of *PTEN* mRNA after knockdown of YTHDC1 could involve either transcriptional induction or increased mRNA stabilization. As YTHDC1 has been reported to regulate RNA stability^24^, we tested this possibility by measuring the half-life of *PTEN* mRNA in YTHDC1-silenced neurons by using actinomycin D (ActD) to inhibit de novo transcription. Knockdown of YTHDC1 in cortical neurons significantly increased the stability of *PTEN* both under normal conditions (Fig. 6B) or early after reoxygenation (3 h) upon ActD treatment (Fig. 6C). Thus, YTHDC1 decreases *PTEN* mRNA stability especially after OGD.

To further investigate the mechanism of YTHDC1-mediated regulation of *PTEN* mRNA stability, we first analyzed whether YTHFC1 binds to *PTEN* mRNA. Indeed, RNA immunoprecipitation (RIP) analysis showed that *PTEN* transcripts were immunoprecipitated by YTHDC1 and this enrichment was increased early after reoxygenation (3 h) (Fig. 6D). Moreover, luciferase reporter assays using a luciferase reporter plasmid with or without 3′-UTR of *PTEN* mRNA in PC12 cells indicated that YTHDC1 inhibited the luciferase activity of *PTEN* 3′-UTR reporter compared to vector cells (Fig. 6E). Therefore, YTHDC1 may decrease *PTEN* mRNA stability via binding its 3′-UTR.

## Discussion

Epigenetic regulation plays an important role in fine-tuning cell function exposing to various stimuli^39,40^. As one of the post-transcriptional epigenetic modifications, the regulation and function of m^6^A modification in response to ischemia is largely unknown. In this study, we found that m^6^A reader YTHDC1 was induced after ischemia. Forced expression or knockdown of YTHDC1 could decrease or increase brain infarct volume, respectively. Mechanistically, YTHDC1 facilitated neuronal survival by promoting Akt phosphorylation through degradation of *PTEN* mRNA.

In this study, we revealed that YTHDC1 might negatively regulate *PTEN* mRNA stability. A previous study showed that transient focal cerebral ischemia leads to increased m^6^A methylation of mRNAs and lncRNAs, and the expression of YTHDF1, YTHDF3 as well as FTO is altered^15^. In combination with the previous study, we demonstrated that m^6^A reader YTHDC1 exerted a crucial role in neuronal survival after cerebral ischemia. Notably, several studies also investigated the role of m^6^A methylation in the survival of cardiomyocytes following ischemic/reperfusion of mouse heart^41,42^. Collectively, these results implicate that global m^6^A methylation are differentially regulated in response to ischemia, and modulating m^6^A methylation through its regulators may provide a way to enhance cell survival.

For the known m^6^A readers, the biological functions of cytoplasmic YTHDF1, YTHDF2, and YTHDF3 have been well studied. The three major readers can modulate cell proliferation, survival, migration, and invasion by regulating RNA stability^17–20^. However, as a major nucleus reader, the role of YTHDC1 in cell survival and proliferation is less clear. In the present study, we showed for the first time that knockdown YTHDC1 could suppress neuronal survival, whereas overexpression of YTHDC1 contributed to neuronal survival. Besides, the induction of YTHDC1 was observed after ischemia, which was consistent with the previous report that the abundance of YTHDF1 and YTHDF2 was also altered after stroke. It is necessary to investigate whether these readers have collaborative or divergent roles in the same cell in response to specific stress.

Here, we demonstrated the involvement of YTHDC1 in *PTEN* mRNA stability control. As a nuclear m^6^A reader, YTHDC1 has been implicated in pre-mRNA splicing^21^. Our data, in particular the regulation of *PTEN* RNA stability by YTHDC1, combined with the results of Shima et al.^24^ suggest that YTHDC1 may have a role in processing mature mRNAs. Given that YTHDC1 can shuttle between the nucleus and cytoplasm, it is plausible to predict that YTHDC1 protein in the cytoplasm may process mature mRNAs, although the detailed molecular mechanism should be further investigated to elucidate whether YTHDC1 decreases *PTEN* mRNA stability through modulating m^6^A modification.

In conclusion, our findings indicate that the m^6^A reader YTHDC1 may serve as an important post-transcriptional regulator which contributes to neuronal survival after cerebral I/R injury. Targeting YTHDC1 provides a potential therapeutic strategy for reducing cerebral I/R injury, although the function of other m^6^A writers, readers, and erasers in ischemic stroke still await further investigation.

## Supplementary information

Supplementary Information

Figure S1
