# Supplementary material for: YTHDC1 mitigates ischemic stroke by promoting Akt phosphorylation through destabilizing PTEN mRNA
Source: Cell Death Dis. 2020 Nov 13;11(11):977. doi: 10.1038/s41419-020-03186-2 (PMC7666223; doi:10.1038/s41419-020-03186-2)
Supplement: Supplementary file 1 — Supplementary Information [file 41419_2020_3186_MOESM1_ESM.docx]

**Supplementary Information**

**Supplementary Materials and Methods**

**Immunoblot analysis**

Cells were lysed with 1× SDS sample buffer (50 mM Tris-HCl, pH 6.8, 100 mM dithiothreitol, 2% SDS, and 10% glycerol) and incubated at 95°C for 10 min. Proteins were separated by SDS-PAGE and transferred to nitrocellulose membranes (GE Healthcare). Membranes were blocked with 5% milk or BSA in Tris buffered saline and Tween 20 for 60 min at room temperature. Proteins were detected with antibodies as described above. Membranes were visualized by immunoblot analysis with the enhanced chemiluminescence detection system (Thermo Fisher Scientific).

**Luciferase reporter assay**

PC12 cells were purchased from the Cell Resource Center of the Shanghai Institute of Biological Sciences of the Chinese Academy of Sciences (Shanghai, China). PC12 cells were plated into 24-well plate and transfected with a mixture of luciferase reporter plasmid containing 3’UTR of *PTEN*, YTHDC1-expression plasmid or vector plasmid, and pRL-TK Renilla luciferase plasmids. Cells were harvested 18-24 h after transfection and luciferase activity was measured with a Dual-Luciferase assay kit (Promega) according to the manufacturer’s instructions.

**Antibodies (Abs) and reagents**

The following antibodies were purchased from Cell Signaling Technology: YTHDC1 Ab (81504); Phospho-p44/42 MAPK (Erk1/2) (Thr202/Tyr204) Ab (9106); p44/42 MAPK (Erk1/2) Ab (4695); PTEN Ab (CST, 9188); [Phospho-Akt (Ser473) Ab (4060S](https://wanlab.lofter.com/post/309234f3_1c607f519)); Phospho-NF-kappa-B p65 (Ser536) Antibody (3031); NF-kappa-B p65 (C22B4) Ab (4764); [Akt (pan 40D4) Ab (2920](https://wanlab.lofter.com/post/309234f3_1c5d806a6)); Cleaved caspase3 (9661); p-mTOR (Ser 2448) (2971); Phospho-Akt (Thr308) Ab (13038). β-actin (bs-0061R) Ab was purchased from Bioss. YTHDF1(17479-1-AP); YTHDF2 (24744-1-AP) were purchased from Proteintech. YTHDC2 (A15004) were purchased from abclonal. Bcl2 (ab196495); Goat anti-rabbit IgG-HRP (ab6721) and Goat anti-mouse IgG-HRP (ab205719) were purchased from Abcam. Actinomycin D (ActD) was brought from MCE.

**Supplementary tables**

**Table 1. Primers for qRT-PCR analysis.**

| **Name** | **Sequence** |
| --- | --- |
| *PTEN*-RT-FP | CAAGATGATGTTTGAAACTAT |
| *PTEN*-RT-RP | CCTTTAGCTGGCAGACCACAA |
| *Actb*-RT-FP | CCAACTGGGACGATATGGAGAAGA |
| *Actb*-RT-RP | AGGTCTCAAACATGATCTGGGTCATC |
| YTHDF1-RT-FP | GACAATGACTTTGAGCCCTACCTTTCT |
| YTHDF1-RT-RP | TTGTCCATAGGTAGTGAGATACGGGA |
| YTHDF2-RT-FP | GAACCTTACTTGAGCCCACAGGCA |
| YTHDF2-RT-RP | CCTCCAGTAGACCAAGCAGCTTCA |
| YTHDF3-RT-FP | GACCTAAAGGGCAAGGAAATAAAGTTT |
| YTHDF3-RT-RP | AAATCCAATGGATGGAGCATAGTAACTA |
| YTHDC2-RT-FP | GACTCAACAATGGCATACCTCAAGTTC |
| YTHDC2-RT-RP | GGTCTTTCCAGACCCAGTTTCTCC |

**Supplementary Figure legends**

**Figure S1. Expression of m^6^A “readers” after ischemia.** (A, B) Immunoblot analysis (A) and Quantitative PCR analysis (B) of YTHDF1, YTHDF2, YTHDF3 and YTHDC2 in tissue extracts if rat brains upon 90-min MCAO followed by various time points of reperfusion. The data are representative or combined from there independent experiments.
