# Supplementary figures and images for: YTHDC1 mitigates ischemic stroke by promoting Akt phosphorylation through destabilizing PTEN mRNA
Source: Cell Death Dis. 2020 Nov 13;11(11):977. doi: 10.1038/s41419-020-03186-2 (PMC7666223; doi:10.1038/s41419-020-03186-2)

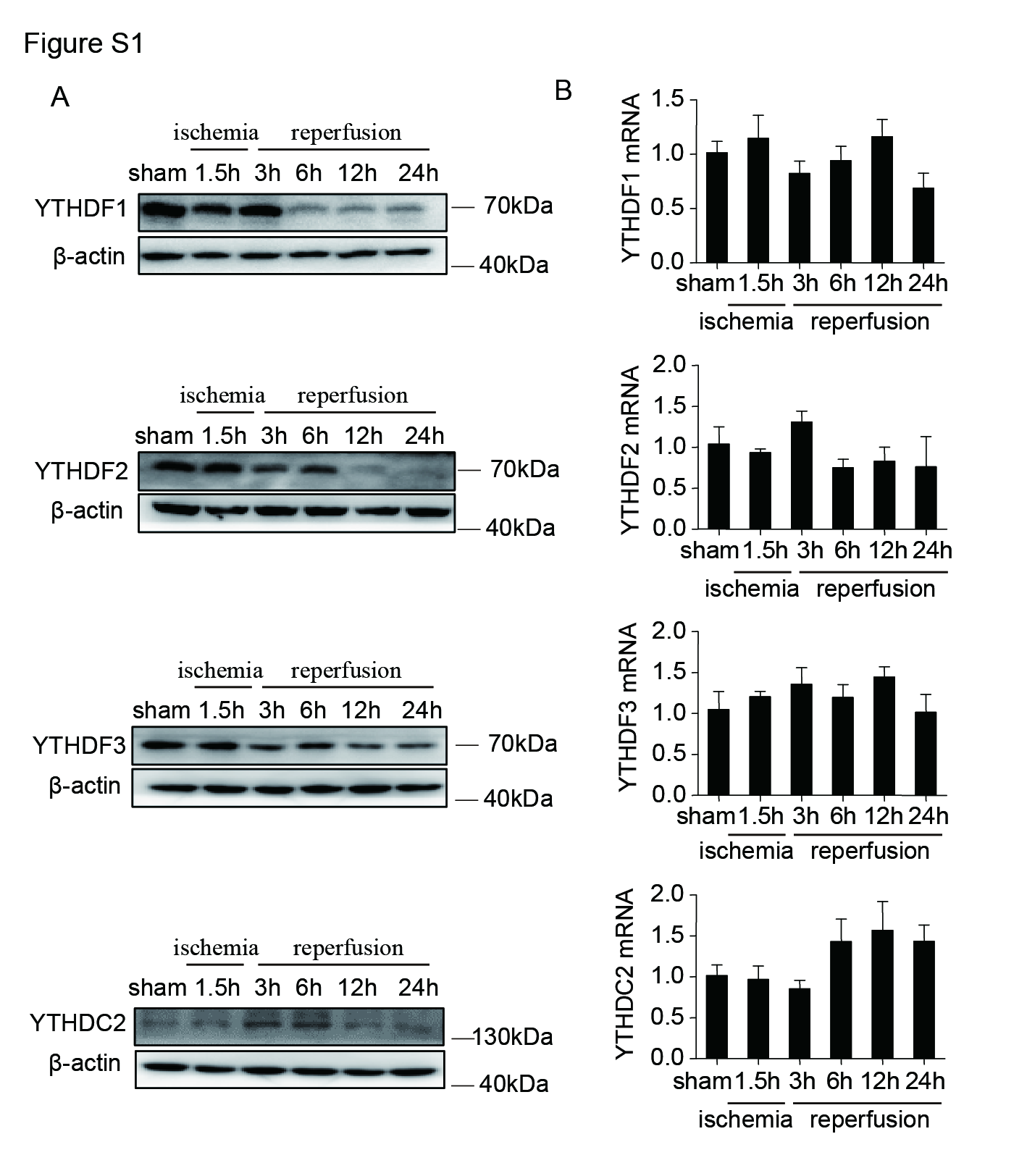

Supplement: Supplementary file 2 — Figure S1 [file 41419_2020_3186_MOESM2_ESM.tif]
